# Establishment of paternal methylation imprint at the *H19/Igf2* imprinting control region

**DOI:** 10.1126/sciadv.adi2050

**Published:** 2023-09-06

**Authors:** Ji Liao, Sangmin Song, Samuel Gusscott, Zhen Fu, Ivan VanderKolk, Brianna M. Busscher, Kin H. Lau, Julie Brind’Amour, Piroska E. Szabó

**Affiliations:** ^1^ Department of Epigenetics, Van Andel Institute, Grand Rapids, MI 49503, USA.; ^2^ Division of Molecular and Cellular Biology, City of Hope Cancer Center, Duarte, CA 91010, USA.; ^3^ Département de Biomédecine Vétérinaire, Université de Montréal, Saint-Hyacinthe, Quebec J2S, Canada.; ^4^ Bioinformatics and Biostatistics Core, Van Andel Institute, Grand Rapids, MI 49503, USA.

## Abstract

The insulator model explains the workings of the *H19* and *Igf2* imprinted domain in the soma, where insulation of the *Igf2* promoter from its enhancers occurs by CTCF in the maternally inherited unmethylated chromosome but not the paternally inherited methylated allele. The molecular mechanism that targets paternal methylation imprint establishment to the imprinting control region (ICR) in the male germline is unknown. We tested the function of prospermatogonia-specific broad low-level transcription in this process using mouse genetics. Paternal imprint establishment was abnormal when transcription was stopped at the entry point to the ICR. The germline epimutation persisted into the paternal allele of the soma, resulting in reduced *Igf2* in fetal organs and reduced fetal growth, consistent with the insulator model and insulin-like growth factor 2 (IGF2)’s role as fetal growth factor. These results collectively support the role of broad low-level transcription through the *H19/Igf2* ICR in the establishment of its paternal methylation imprint in the male germ line, with implications for Silver-Russell syndrome.

## Teaser

Genetic experiments find that paternal imprint establishment requires broad low-level transcription in prospermatogonia.

## INTRODUCTION

Imprinted genes often cluster in domains and have a special expression pattern of being transcribed from one parental chromosome only, the one inherited from the father, or the one inherited from the mother. The two chromosomes carry different epigenetic markings in the form of DNA methylation at the imprinting control region (ICR) that regulates parental allele–specific expression of genes along the domain. Those imprinted germline differentially methylated regions (gDMRs) originate in the male or female germ lines and are carried into the zygote from the sperm or the oocyte. DMRs are later maintained in the paternally (PAT) or maternally (MAT) inherited chromosome during development of the soma. One outstanding question is why PAT and MAT gDMR establishment differ in the male and female germ lines.

**Fig. 1.**
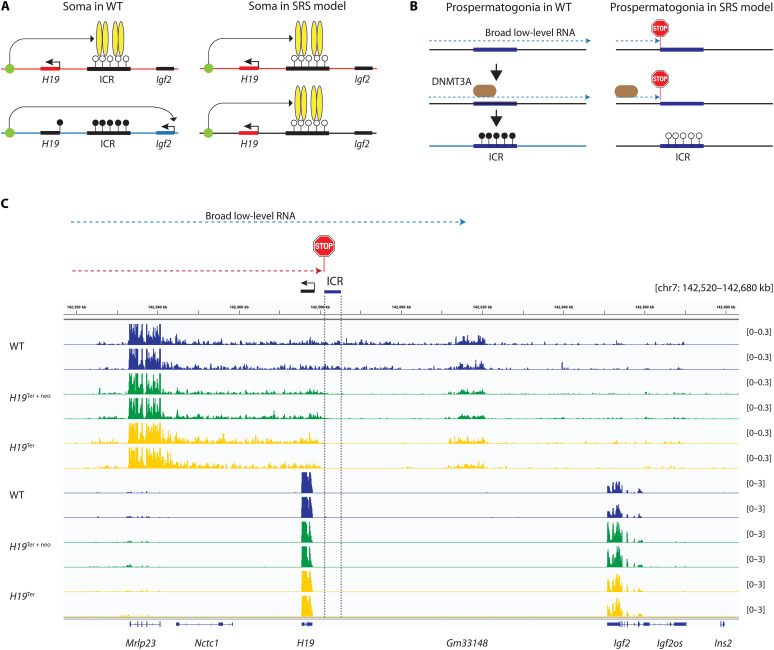
Testing the function of broad low-level transcription at the *H19/Igf2* ICR. (**A**) Insulator model. CTCF molecules (yellow ovals) bind in the unmethylated ICR allele in the chromosome inherited from mother and block *Igf2* from the shared enhancers (green dot). Methylation of the ICR inherited from the father via the sperm prevents CTCF binding and thus allows activation of *Igf2*. The panel to the right shows the expected aberrant gene expression pattern when the ICR is not methylated properly in the paternal chromosome, as in the case of the majority of SRS patients. (**B**) Hypothesis: Paternal methylation imprint is established in prospermatogonia by de novo methyltransferase DNMT3A in response to broad low-level transcription that runs through the ICR. The right panel depicts the expected outcome when transcription is terminated before entering the ICR in prospermatogonia. (**C**) Transcription across the ICR is terminated by the knock-in terminator cassette. Genome browser view of normalized total RNA sequencing (RNA-seq) bigwig files in the forward (top six lanes) and reverse (bottom six lanes) directions. Biological replicates of 15.5 dpc purified prospermatogonia from wild-type (WT), *H19*^Ter+neo^/*H19*^Ter+neo^, and *H19*^Ter^/*H19*^Ter^ homozygous fetuses. The genes and the *H19/Igf2* ICR and the insertion site for the termination cassette are marked.

Mouse genetic studies found that the reciprocal maternal and paternal allele–specific expression of the *H19* and *Igf2* genes depends on an ICR, which behaves as a maternal allele–specific CTCF-mediated insulator in somatic cells (Fig. 1A). DNA methylation inherited from the sperm inhibits insulation in the paternal allele. A major mystery in the imprinting field is what specifies DNA methylation at this PAT DMR in the male germ line. This question is relevant to Silver-Russell syndrome (SRS; OMIM 180860) (*1*), an imprinting disorder, manifesting in fetal and postnatal growth retardation and related symptoms. Loss of methylation is detected in the ICR1 that controls the *H19/IGF2* domain in about 65% of SRS cases (*2*, *3*). The cause of this epigenetic deficiency, however, is unknown. It may involve the process of imprint establishment—de novo methylation of ICR1 in the male germ line.

Our genome-wide deep total RNA-sequencing (RNA-seq) experiments (*4*) revealed that broad low-level transcription runs through the *H19/Igf2* ICR specifically in 15.5 days postcoitum (dpc) mouse prospermatogonia (fig. S1), at the time when the establishment of PAT gDMRs, such as the *H19/Igf2* ICR, is initiated (*4*–*6*). We hypothesized that this transcription through the ICR is required for targeting de novo methylation to the ICR in prospermatogonia (Fig. 1, A and B) and tested its role using mouse genetics.

## RESULTS

### Insertion of an RNA terminator cassette by gene targeting truncates the prospermatogonia-specific transcript

We carried out a gene targeting experiment in mouse embryonic stem (ES) cells. We inserted a transcription terminator cassette (Ter) (*7*) to truncate the fetal male germ cell–specific broad low-level transcript (*4*) before entering the *H19/Igf2* ICR sequences (fig. S2) (*8*, *9*). 

To test whether the insertion results in truncation of the broad low-level transcription, we purified prospermatogonia from 15.5 dpc *H19*^Ter^/*H19*^Ter^ and *H19*^Ter+neo^/*H19*^Ter+neo^ homozygous fetuses. All these fetuses also carried an Oct4–enhanced green fluorescent protein (EGFP) transgene, crossed in from the TgOG2 transgenic mouse (*10*) to facilitate fluorescence-activated cell sorting (FACS) of EGFP^+^ germ cells out of fetal testes. We carried out a deep total RNA-seq experiment as earlier (*4*). We found that the broad low-level transcript stopped inside the terminator cassette before entering the ICR in *H19*^Ter^/*H19*^Ter^ prospermatogonia or before entering the Pgkneo cassette in *H19*^Ter+neo^/*H19*^Ter+neo^ prospermatogonia (fig. S3). We also noted that the *H19*^Ter+neo^/*H19*^Ter+neo^ prospermatogonia exhibited no transcription across the Pgkneo cassette in the forward direction (fig. S3). No transcript exited into neighboring sequences out of the cassettes in either forward or reverse direction (fig. S3). In response to the terminator insertion, transcription was diminished across the ICR in the prospermatogonia of *H19*^Ter+neo^/*H19*^Ter+neo^ and *H19*^Ter^/*H19*^Ter^ fetuses (Fig. 1C).

### Broad low-level transcription across the ICR is required for paternal methylation imprint establishment

**Fig. 2.**
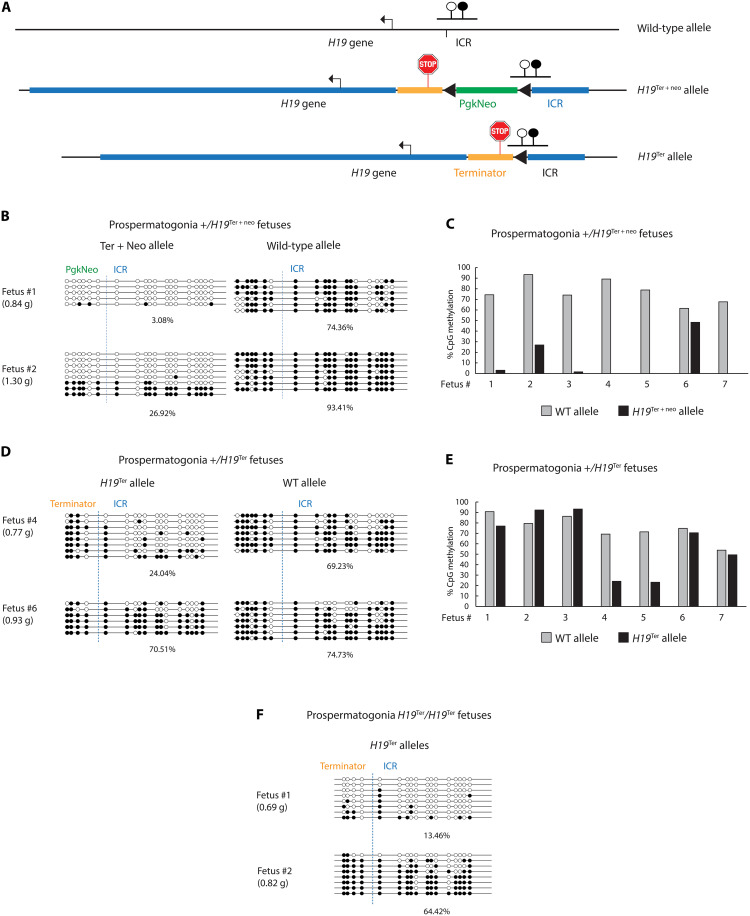
De novo DNA methylation of the *H19/Igf2* ICR requires transcription through the ICR in prospermatogonia. (**A**) Schematic map of the bisulfite sequencing regions in the WT and mutant alleles. (**B** to **F**) Results of bisulfite sequencing in EGFP^+^ FACS-purified prospermatogonia from 17.5 dpc fetuses. Open and closed circles indicate unmethylated and methylated CpGs, respectively. (B) Examples of *H19*^+^/*H19*^Ter+neo^ fetuses. Dashed vertical lines separate the ICR sequences from the flanking regions. The weight of each fetus is indicated to the left. (C) Individual *H19*^+^/*H19*^Ter+neo^ fetuses at 17.5 dpc obtained from three litters of three mutant males out of three independent founders. The percentage of methylated CpGs of 13 CpGs in the ICR fragment is plotted in the WT and knock-in allele. (D) Examples of *H19*^+^/*H19*^Ter^ fetuses. (E) Individual *H19*^+^/*H19*^Ter^ fetuses obtained from three litters of two males out of two founder males. (F) Examples of DNA methylation in prospermatogonia of the *H19*^Ter^/*H19*^Ter^ fetuses.

We performed bisulfite DNA sequencing in purified prospermatogonia from individual 17.5 dpc heterozygous *H19*^+^/*H19*^Ter+neo^ and *H19*^+^/*H19*^Ter^ fetuses (Fig. 2A). Note that the allele inherited from the mother is written first in each genotype. In these samples, DNA methylation of the wild-type (WT) allele has reached a high level of DNA methylation by 17.5 dpc as expected (*11*). However, CpG methylation of the mutant allele was lower than the WT allele in prospermatogonia of each *H19*^+^/*H19*^Ter+neo^ fetus (Fig. 2, B and C). CpG methylation was variable in the *H19*^Ter+neo^ allele between fetuses and even more so in the *H19*^Ter^ allele, which was hypomethylated in two of seven fetuses (Fig. 2, D and E). In *H19*^Ter^/*H19*^Ter^ prospermatogonia, CpG methylation of the ICR was 64 and 13% (Fig. 2F), again a reduction compared to the expected levels (*11*). In the RNA-seq data, the *H19*^Ter+neo^/*H19*^Ter+neo^ mice showed some low-level transcription through the ICR (Fig. 1C), which appears to be a reinitiation after a complete stop has occurred in the terminator cassette (fig. S3B). According to the model (Fig. 1B), one would predict that the *H19*^Ter+neo^ allele should have more DNA methylation compared to that of *H19*^Ter^ allele, where a complete lack of transcription was observed (Fig. 1C). The opposite result was found (Fig. 2, C and E), perhaps because the Pgkneo cassette has an effect on ICR methylation in the *H19*^Ter+neo^ allele. Nevertheless, these results collectively demonstrate that DNA methylation establishment at the *H19/Igf2* ICR does not occur properly in the absence of the prospermatogonia-specific broad, low-level transcription across the ICR sequences.

### Aberrant methylation imprint establishment results in reduced growth in the offspring

**Fig. 3.**
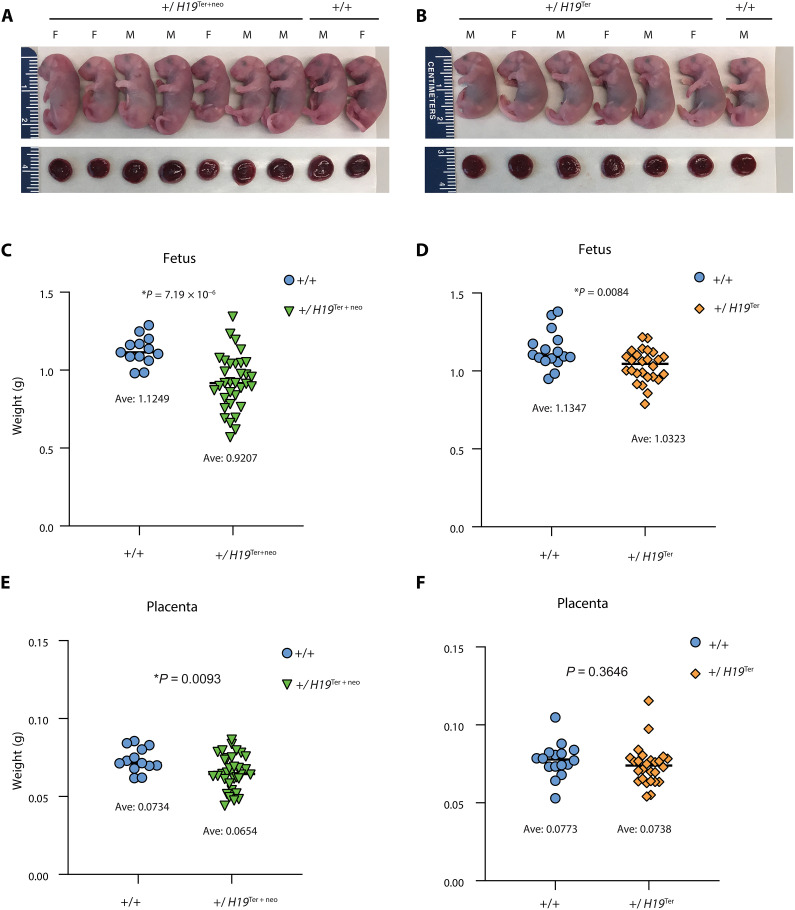
Offspring derived from epimutant prospermatogonia exhibit variably reduced fetal and placental growth. (**A**) Photographs of uterus-mate 18.5 dpc *H19*^+^/*H19*^Ter+neo^ and *H19*^+^/*H19*^+^ fetuses are displayed with the corresponding placentas. (**B**) Photographs of uterus-mate 18.5 dpc *H19*^+^/*H19*^Ter^ and *H19*^+^/*H19*^+^ fetuses and corresponding placentas. The sex of each fetus is marked as female (F) and male (M). (**C**) Weight distribution of *H19*^+^/*H19*^Ter+neo^ fetuses (*n* = 33) and control *H19*^+^/*H19*^+^ siblings (*n* = 13) from seven litters. (**D**) Weight distribution of *H19*^+^/*H19*^Ter^ fetuses (*n* = 26) and control *H19*^+^/*H19*^+^ siblings (*n* = 16) from seven litters. (**E**) Weight distribution of the placentas of the *H19*^+^/*H19*^Ter+neo^ fetuses (*n* = 33) and *H19*^+^/*H19*^+^ siblings (*n* = 13) from seven litters as above. (**F**) Weight distribution of the placentas of *H19*^+^/*H19*^Ter^ fetuses (*n* = 26) and control *H19*^+^/*H19*^+^ siblings (*n* = 16) from seven litters as above. The average value is shown as a horizontal line. The significance of difference between genotypes (*P* value calculated by two-tailed *t* tests) is included.

On the basis of the insulator model (Fig. 1A) (*12*–*15*), we hypothesized that inheriting a reduced ICR methylation from the male germline in the paternal allele results in reduced growth in the mutant fetuses. In this scenario, the *Igf2* gene, encoding a fetal growth factor, is expected to be insulated in both parental alleles. The weight of *H19*^+^/*H19*^Ter+neo^ and *H19*^+^/*H19*^Ter^ fetuses and their placentas was variably reduced at 18.5 dpc (Fig. 3, A to F). The weight reduction of *H19*^+^/*H19*^Ter+neo^ fetuses and placentas, and *H19*^+^/*H19*^Ter^ fetuses was statistically significant. To find out whether the reduced growth lasts beyond fetal life, we measured the weight of weanling pups and adults (fig. S4). The weight of the mutant *H19*^+^/*H19*^Ter+neo^ pups, males and females, was significantly lower compared with WT siblings. The number of *H19*^+^/*H19*^Ter+neo^ mutant pups was much less than expected at weaning. This bias likely resulted from early postnatal lethality, because of the 17 dead pups found during the first 3 days of birth, 12 were *H19*^+^/*H19*^Ter+neo^ and 5 were WT. The *H19*^+^/*H19*^Ter+neo^ but not *H19*^+^/*H19*^Ter^ weanling pups and adults showed a broader distribution of weights than their WT siblings at 3 and 8 weeks. The weight of *H19*^+^/*H19*^Ter+neo^ adults was also reduced compared to WT littermates, and, in males but not females, the difference was significant. We noticed that the WT siblings of *H19*^+^/*H19*^Ter^ mice, both females (*P* = 8.2959 × 10^−7^) and males (*P* = 2.1692 × 10^−5^), were significantly smaller at weaning and at 8 weeks of age (*P* = 0.0010 and *P* = 0.0576, respectively) than the WT siblings of *H19*^+^/*H19*^Ter+neo^ mice. The reason for this difference is not known. It did not initiate at fetal stage, as those WT fetuses and their placentas were not significantly different between crosses (*P* = 0.8040 and *P* = 0.2896). The *H19*^+^/*H19*^Ter^ mice and *H19*^+^/*H19*^Ter+neo^ mutant mice were not different from each other. These results collectively suggest that defective methylation establishment in prospermatogonia has a lasting phenotypic effect in the next generation, as paternal inheritance of the Ter insertion results in reduced fetal and placental growth in both mutant lines and reduced postnatal weight in the *H19*^+^/*H19*^Ter+neo^ and perhaps also the *H19*^+^/*H19*^Ter^ mutant mice.

### The variable methylation persists from epimutant prospermatogonia to the offspring

**Fig. 4.**
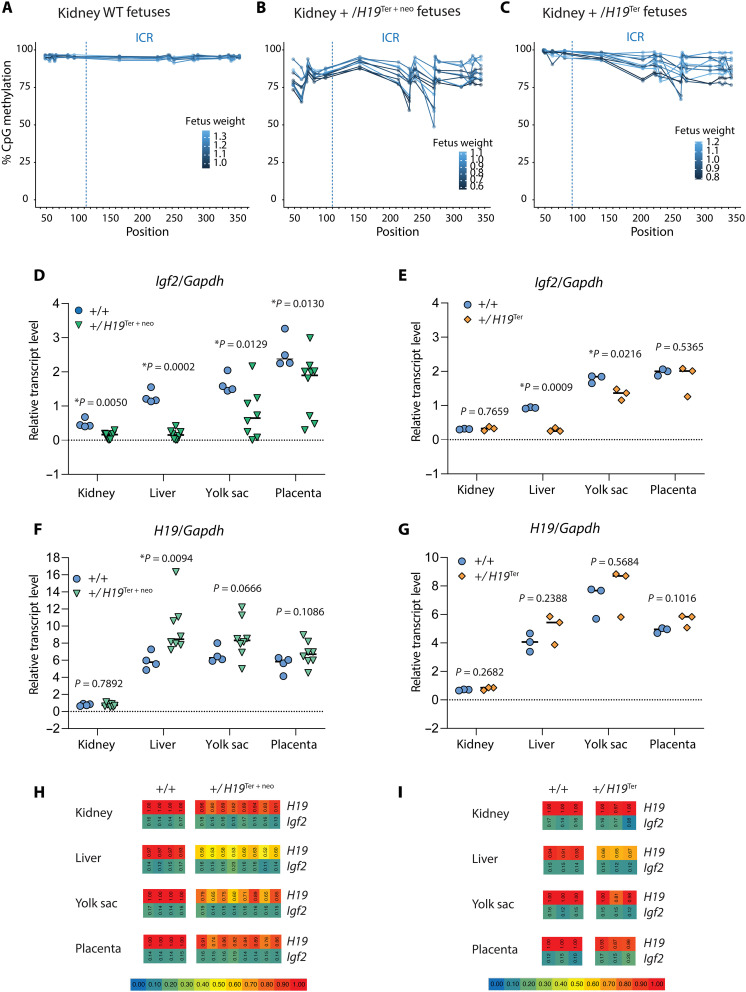
ICR hypomethylation is transmitted from the germline to the offspring and results in aberrant expression of the *Igf2* and *H19* imprinted genes. (**A** to **C**) Multiplex bisulfite sequencing results shown in the PAT inherited allele in the kidney of 18.5 dpc fetuses (A to C). Percentage of methylation is plotted at each CpG in the ICR and flanking regions (left of the dotted line). (A) WT *H19*^+^/*H19*^+^ (*n* = 8), (B) *H19*^+^/*H19*^Ter+neo^ (*n* = 11), and (C) *H19*^+^/*H19*^Ter^ (*n* = 11). (**D** to **G**) Quantitative reverse transcription polymerase chain reaction (PCR) in four fetal organs at 18.5 dpc. Experimental crosses were carried out as in Fig. 3. The average value for each organ is marked with a horizontal bar (*P* value by two-tailed *t* tests). (D) *Igf2* in *H19*^+^/*H19*^Ter+neo^ fetuses. (E) *Igf2* in *H19*^+^/*H19*^Ter^ fetuses. (F) *H19* in *H19*^+^/*H19*^Ter+neo^ fetuses. (G) *H19* in *H19*^+^/*H19*^Ter^ fetuses. (**H** and **I**) Sequenom allelotyping of allele-specific transcription in four organs of mutant and control 18.5 dpc fetuses. The ratio of maternal/(maternal + paternal) allele for the *H19* and *Igf2* imprinted genes is displayed by the color scale. Genotypes are shown at the top. (H) *H19*^+^/*H19*^Ter+neo^ fetuses. (I) *H19*^+^/*H19*^Ter^ fetuses.

To test whether the reduced DNA methylation establishment in prospermatogonia persists into the PAT inherited allele of the soma in the next generation, we carried out multiplex indexed bisulfite sequencing in the kidney of *H19*^+^/*H19*^Ter+neo^ and *H19*^+^/*H19*^Ter^ fetuses at 18.5 dpc. Upon paternal transmission, both the *H19*^Ter+neo^ and *H19*^Ter^ alleles were variably hypomethylated compared to the WT allele in control fetuses (Fig. 4, A to C). Lighter fetuses displayed lower level of DNA methylation at the ICR (Fig. 4, B and C), suggesting that the variable ICR hypomethylation inherited from the mutant male germline affects growth in the mutant fetuses, and the severity of growth defect corresponds to the severity of methylation defect. These results demonstrate that the variably hypomethylated state of the prospermatogonia is passed on to the offspring in the paternal allele resulting in variable growth phenotype. It is also interesting that prospermatogonia with the lower ICR DNA methylation was obtained from smaller fetuses (Fig. 2, B, D, and F), suggesting heritability of the variable methylation between generations.

### Aberrant imprinted gene expression is detected in the offspring out of epimutant prospermatogonia

The insulator model (Fig. 1A) predicts that hypomethylation of the ICR in the mutant paternal allele results in biallelic insulation of the *Igf2* promoters from the enhancers (*16*) that lie downstream of *H19*, as observed in genetic SRS model mice (*17*–*19*). *Igf2* levels were greatly and significantly reduced in the kidney, liver, yolk sac, and placenta organs of 18.5 dpc *H19*^+^/*H19*^Ter+neo^ fetuses and in the liver and yolk sac of *H19*^+^/*H19*^Ter^ fetuses compared with WT fetuses (Fig. 4, D and E). Because spreading of the ICR hypermethylation is required for silencing the *H19* promoter during embryo development (*11*, *20*), we expected an up-regulation of *H19* RNA in the mutant fetuses that inherit hypomethylated ICR from their father (Fig. 1A). *H19* levels were variably increased in the liver and yolk sac and placenta of 18.5 dpc *H19*^+^/*H19*^Ter+neo^ and *H19*^+^/*H19*^Ter^ fetuses, and these differences were statistically significant in the liver of *H19*^+^/*H19*^Ter+neo^ fetuses (Fig. 4, F and G). Derepression of *H19* in the paternal allele should result in a shift from maternal allele–specific to biallelic expression of *H19.* To quantify parental alleles, we performed Sequenom allelotyping experiments as earlier (*17*, *18*). The ratio of maternal/(maternal + paternal) allele of *H19* was reduced in the liver, yolk sac, and placenta and slightly in the kidney of 18.5 dpc *H19*^+^/*H19*^Ter+neo^ fetuses compared to control *H19*^+^/*H19*^+^ fetuses (Fig. 4H). Relaxation of H19 imprinting was also detected in the liver of *H19*^+^/*H19*^Ter^ fetuses compared with control *H19*^+^/*H19*^+^ fetuses (Fig. 4I). Parental allele–specific expression of imprinted genes was not affected beyond the *H19/Igf2* domain along distal chr7 (figs. S5 and S6). These results demonstrate that compromised methylation imprint establishment in prospermatogonia has severe consequences to the expression of both *H19* and *Igf2* imprinted genes upon paternal inheritance.

## DISCUSSION

This work supports a role of transcription through the ICR in the establishment of de novo DNA methylation at the *H19-Igf2* gDMR in prospermatogonia: (i) The RNA-terminator cassette prevents the broad low-level transcript from entering the ICR; (ii) DNA methylation establishment is reduced at the ICR in fetal male germ cells; (iii) DNA hypomethylation is inherited to the offspring in the paternal allele; (iv) ICR hypomethylation in the soma results in biallelic insulation and causes reduced *Igf2* and biallelic *H19* transcription; and (v) reduced *Igf2* levels result in fetal growth retardation.

The stochastic epigenetic effects in this mouse model resemble SRS cases with epigenetic molecular features. We found that DNA methylation of the ICR was partially reduced in both mutant lines. Although the terminator cassette stopped the broad low-level transcript before entering the ICR or the Pgkneo cassette (fig. S3), it did not prevent the restarting of the transcript in the ICR (albeit at a much lower level), which, if it occurs in one germ cell but not in another, can underlie the stochastic nature of the effect on DNA methylation establishment. A partial ICR hypomethylation in the soma allows partial gain of insulation and partial loss of *Igf2* transcription with partially reduced fetus and placenta weight. DNA methylation was established at a variable degree in the prospermatogonia of different mutant fetuses and resulted in variable size offspring out of mutant germ cells. Smaller fetuses had more profound hypomethylation at the ICR in their perinatal prospermatogonia. This model provides a useful experimental system for studying the heritability of epigenetic states between generations.

It is interesting to compare the mechanisms of imprinted gDMR establishment between the female and male germ lines. The de novo methyltransferase DNMT3A, its cofactor DNMT3L (*21*–*26*), and the H3K4 demethylase KDM1B (*27*, *28*) are required for establishing maternal gDMRs in growing oocytes. In addition, transcription initiated from oocyte-specific alternative promoters is required along the MAT methylated gDMRs for inducing DNA methylation in growing oocytes (*25*, *29*–*34*), in an analogous mechanism to gene body methylation (*35*), where the SETD2-dependent H3K36me3 is required (*36*) to attract the PWWP domain of DNMT3B (*37*, *38*). In fetal male germ cells, transcription that runs across paternal gDMRs is different from that found in growing oocytes. It is very broad, spans hundreds of kilobases, and occurs at such a low level in prospermatogonia that can only be detected by very deep sequencing of total RNA. It is a genome-wide feature of prospermatogonia (*4*). In those cells, the role of SETD2 is dispensable, but H3K36me2 methyltransferase NSD1 is required for establishing DNA methylation at three paternal DMRs, the *H19*-*Igf2*, *Dlk1*-*Gtl2*, and part of the *Rasgrf1* DMR (*39*). NSD1-mediated H3K36me2 has been shown in mouse mesenchymal stem cells and ES cells to target the PWWP domain of DNMT3A and DNA methylation to intergenic regions in the genome (*40*, *41*). When we carried out chromatin immunoprecipitation sequencing (ChIP-seq) experiments in the 15.5 dpc *H19^Ter^*/*H19*^Ter^ mutant prospermatogonia (fig. S7), we detected no difference in H3K36me2 (or H3K36me3 and H3K27me3) levels along the *H19/Igf2* gDMR or surrounding sequences. This finding suggests that H3K36me2 affects de novo DNA methylation at those sequences independent of broad low-level transcription. Future experiments will test whether the prospermatogonia-specific transcripts recruit DNMT3A and perhaps NSD1 to other paternal gDMRs. Our RNA-seq experiment detected specific transcription through each PAT methylated imprinted gDMR (*4*). Therefore, similar to the *H19/Igf2* gDMR, paternal imprint establishment of the *Dlk1*-*Gtl2* and perhaps also at one-half of the *Rasgrf1* DMR that depends on NSD1 (*39*) may require transcription-through. The other part of the *Rasgrf1* gDMR has a special Piwi-interacting RNA–mediated mechanism for methylation establishment (*42*) and is methylated by DNMT3C (*43*). Similarly, we predict that PAT imprinted gDMRs will attain imprinting in the male germ line only when introduced into genomic regions that exhibit broad low-level transcription in prospermatogonia. When the *H19/Igf2* ICR was inserted upstream of the *Afp* promoter (*44*) that has no transcription through (fig. S8), it failed DNA methylation establishment in the germline, although it still became methylated in the embryo. If the transgenic constructs also carry the elements necessary for methylation maintenance, such as the ZFP57 recognition sites (*45*–*47*), then those transgenes will behave imprinted in the offspring. When the human *H19/IGF2* ICR1 was knocked into the mouse locus, it showed incomplete (~50%) methylation in the sperm and a further reduced DNA methylation by the two-cell stage and became unmethylated in the paternal allele of the offspring (*48*). Therefore, the human ICR1 sequences are not capable of imprint maintenance in the mouse. It will be interesting to examine whether transcription runs through the human ICR1 and whether incomplete DNA methylation establishment occurs at those sequences in mouse prospermatogonia.

The limitation of this work is that we cannot fully address the heritability of the stochastic methylation aberrations. We cannot trace germ cell methylation pattern directly into the soma in a specific chromosome because if we examine one physical chromosome copy in the germ line, then it will not give rise to offspring. Another limitation is that, although using the rabbit β-globin RNA truncation cassette (*7*) has been a well-accepted genetic test for the function of transcription in DNA methylation establishment at MAT imprinted gDMRs (*29*, *31*, *32*, *34*), we cannot completely exclude the possibility that altered methylation at the *H19/Igf2* paternal gDMR is a consequence of the insertional mutation by an unknown mechanism and not a consequence of the reduced read through transcription by specifically terminating the transcript.

In summary, we have uncovered the mechanism that instructs methylation imprint establishment at the specific sequences of the *H19/Igf2* ICR at the endogenous location in fetal male germ cells, and by manipulating this mechanism, we generated an epigenetic SRS model resembling the majority of human SRS cases (*2*), in that it has partial stochastic ICR hypomethylation. By providing fundamental knowledge on imprint establishment at the gDMR and developing a mouse epigenetic model, this work supports future studies aimed at ameliorating microsomia in the epigenetic subset of SRS. This SRS model has no genetic mutation inside the ICR and displays milder phenotypes than our previous genetic SRS models, where the ICR was replaced by the chicken β-globin insulator (*17*, *18*). Errors in establishment rather than methylation maintenance may be more relevant in the etiology of ICR1 hypomethylation in patients with SRS, as no evidence was found for ZFP57 mutations in SRS epimutations (*49*). The methylation deficit at the ICR is consistent with a partial reduction of the *Igf2* transcript and insulin-like growth factor 2 (IGF2) protein levels in SRS fetuses, as well as the fetal growth retardation and microsomia symptoms. This SRS model will allow epigenetic, genetic, or pharmacological corrections of growth retardation in SRS of epigenetic origin. In these cases, therapeutically correcting IGF2 signaling at the fetal stage could be beneficial (*50*).

## MATERIALS AND METHODS

All animal experiments were performed according to the National Institutes of Health Guide for the Care and Use of Laboratory animals, with Institutional Care and Use Committee–approved protocols at Van Andel Institute (VAI). Female and male uterus-mate fetuses from multiple litters were used as biological replicates in the experiments that compare RNA levels, allelic expression, DNA methylation, and weight between the genotypes. Fetus, newborn, and weanling numbers are provided in the figures.

### Mouse lines

The *H19*^Ter+neo^ and *H19*^Ter^ mouse models were generated using gene targeting in 129SI ES cells as we did earlier (*11*, *18*). After blastocyst injection of two positive ES cell clones, we obtained 13 chimeras (four females and nine males) from one ES cell line. To obtain the *H19*^Ter+neo^ founders and rescue the anticipated PAT inherited growth phenotype similar to the genetic rescue of another SRS model (*50*), three male chimeras were crossed to females in the 129S1 background that carried point mutations in the four CTCF binding sites in the *H19/Igf2* ICR (*11*). The mutation was subsequently maintained through the female germ line to avoid the disadvantage of small paternal mutants in the breeding litters; the breeding pairs were set up by crossing mutant females with WT 129S1 males. This breeding practice provided the experimental heterozygous fathers. To obtain the *H19*^Ter^ line, the Pgkneo selection cassette was removed by crossing the *H19*^Ter+neo^ mutant mice with the Hprt-Cre expressing 129S1/Sv-Hprt^tm1(CAGcre)Mann/J^ transgenic mouse line (*51*). The *H19*^Ter^ line mutation was also maintained through the female germ line. The JF1/MsJ inbred strain from The Jackson Laboratory (JAX 003720) was used for distinguishing parental alleles due to the presence of single-nucleotide polymorphisms (SNPs) in its genome compared with our mouse model. For germ cell collection, the *H19*^Ter+neo^ and *H19*^Ter^ lines were crossed with the TgOG2 transgenic mouse B6;CBA-Tg^(Pou5f1-EGFP)2Mnn^ that allows purification of prospermatogonia by FACS (*10*).

### Southern blot hybridization

Ten micrograms of genomic DNA from the polymerase chain reaction (PCR)–positive ES clones were digested with restriction enzyme, then separated by 0.8% agarose gel electrophoresis, and transferred to Amersham Hybond nylon membrane (RPN1210B, GE Healthcare), and then Southern blot hybridization experiments were performed using dioxigenin-labeled probes. Visualization was done by exposing to x-ray film for 30 min. PCR primers for generating these probes were as follows: probe 1 (outside short arm): 5′ GCA ATC CGT TTT AGG ACT GCG ATG TAC GAG AC 3′ and 5′ GCT ACA TTC ACA CGA GCA TCC AGG AGG C 3′ to detect an 8.7-kb WT and 11.5-kb mutant Bam HI band; probe 2 (neo cassette): 5′ CGG CAG GAG CAA GGT GAG ATG AC 3′ and 5′ CGC TTG GGT GGA GAG GCT ATT CG 3′; probe 3 (inside long arm): 5′ CAG AGA GCA GCA GAG AAG TGT TAG CTC TTT GGG 3′ and 5′ GTA AGT GTC TGT CCC GCT CGT GGT CA 3′ to detect a 4-kb WT and 7-kb mutant Hind III band.

### PCR genotyping of the mutant ES cells and mice

Oligonucleotide primers ON39 (5′ TCA GTG AGA TGA GTT GGG AGC ACT ACC A 3′), ON40 (5′ AGA ACA ATC AAG GGT CCC CAA ACT CAC C 3′), and ON41 (5′ GAG CGT GCA GGG CAC TTA CAC C 3′) confirmed the correct recombination along the long arm by amplifying a 14-kb fragment in the WT versus a 12-kb fragment in the mutant in 500 ng of ES cell DNA using the LA Taq DNA Polymerase (RR002M, Takara) in 1× LA PCR buffer with MgCl_2_ and using PCR cycles at 94°C for 1 min, (94°C, 30 s, 70°C, 15 m) for 35 cycles and 72°C for 10 min. Mutant bands were resolved in a 0.8% agarose gel. Mouse tail tip samples were collected at weaning time, boiled in 100 μl of tail digestion buffer (*52*) (25 mM NaOH and 2 mM EDTA) at 95°C for 30 min, and dissociated by vortexing. Tail neutralization buffer [100 μl; 40 mM tris-HCl (pH 4.3)] was added to stop the digestion. PCR were performed with 1 μl of digested tail complex in 10 μl of total volume including 2× GoTaq Green Master Mix (M7122, Promega) using the PCR cycles: 95°C for 2 min, (95°C, 30 s, 65°C, 45 s, 72°C, 45 s) for 40 cycles and 72°C for 5 min. Mutant bands were resolved in a 2% agarose gel. Oligonucleotide primers ON34 with ON37 detected the *H19*^Ter+neo^ allele [199 base pairs (bp)]; Neo47 with Neo48 also detected the *H19*^Ter+neo^ allele (293 bp); and ON36 with ON37 detected the *H19*^Ter^ allele (371 bp). The primer sequences were the following: ON34, 5′ GGG GCT GCT AAA GCG CAT GC 3′; ON36, 5′ AAC AGC CCC CTG CTG TCC A 3′; and ON37, 5′ AAG CTT TGA GTA CCC CAG GTT CAA CAA AGG 3′.

### Purification of fetal male germ cells

To obtain homozygous mutant male fetal germ cells, *H19*^Ter+neo^*/H19*^Ter+neo^ or *H19*^Ter^/*H19*^Ter^ females that carry the *Pou5f1* promoter-driven EGFP transgene from the TgOG2 (*10*) mouse line were crossed with males of the same genotype. To obtain heterozygous male fetal germ cells, TgOG2 (*10*) females were crossed with *H19*^Ter+neo^ or *H19*^Ter^ males. The fetal testicles were dissected, and a single-cell suspension was prepared by trypsin digestion and trituration as described earlier (*10*). Up to eight testes were digested in 150 μl of 0.25% trypsin (including 0.5% bovine serum albumin) at 36°C for 15 min, tapping gently every 5 min. The trypsin reaction was stopped by adding 450 μl of 20% fetal bovine serum in M2 medium (catalog no. M7167, Sigma-Aldrich). The EGFP^+^ germ cells were purified by flow cytometry using a Beckman Coulter MoFlo Astrios sorter.

### Construction and sequencing of directional total RNA-seq libraries

Total RNA was isolated using RNA-Bee reagent (Tel-Test) from FACS sorted 15.5 dpc male germ cells, followed by deoxyribonuclease I treatment. Libraries were prepared by the VAI Genomics Core from 500 ng of total RNA using the KAPA RNA HyperPrep Kit (Kapa Biosystems, Wilmington, MA, USA). Ribosomal RNA material was reduced using the QIAseq FastSelect –rRNA HMR Kit (QIAGEN, Germantown, MD, USA). RNA was sheared to 300 to 400 bp. Before PCR amplification, cDNA fragments were ligated to IDT for Illumina TruSeq UD indexed adapters (Illumina Inc., San Diego, CA) and amplified with 8 cycles of PCR. Quality and quantity of the finished libraries were assessed using a combination of Agilent DNA High Sensitivity chip (Agilent Technologies Inc.) and QuantiFluor dsDNA System (Promega Corp., Madison, WI, USA). Individually indexed libraries were pooled, and 50-bp, paired-end sequencing was performed on an Illumina NovaSeq 6000 sequencer to an average depth of 100 million raw paired reads per transcriptome. Base calling was done by Illumina RTA3, and output of noncrystallographic symmetry was demultiplexed and converted to FastQ format with Illumina Bcl2fastq v1.9.0.

### RNA-seq analysis

Adaptor sequences and low-quality reads were trimmed using TrimGalore (*53*) v0.6.0. (https://github.com/FelixKrueger/TrimGalore). Trimmed reads were aligned to the mm10 reference genome using STAR (*54*) v2.7.8 with parameter “--quantMode GeneCounts”. Module bamCoverage in deepTools (*55*) was used to generate bigwig coverage tracks from bam files that were generated from STAR aligner. In addition, RNA-seq reads were aligned to chromosome 7 that included the 2906-bp-long inserted cassette sequences at 142,580,156 using STAR; bigwig files were generated using bamCoverage from deepTools.

### Ultralow-input native ChIP-seq of prospermatogonia

Male germ cells were resuspended in nuclear isolation buffer (Sigma-Aldrich), flash-frozen in liquid nitrogen and stored at −80°C until use. Chromatin was prepared from 100,000 cells and divided into four fractions for H3K36me2, H3K36me3, and H3K27me3 for ultralow-input native ChIP-seq (*56*). Briefly, the chromatin was fragmented by micrococcal nuclease digestion (NEB) for 7.5 min at 37°C, diluted in native ChIP buffer [20 mM tris-HCl (pH 8.0), 2 mM EDTA, 150 mM NaCl, and 0.1% Triton X-100] containing 1 mM phenylmethylsulfonyl fluoride (Sigma-Aldrich) and EDTA-free protease inhibitor cocktail (Roche). Each chromatin fraction was incubated overnight at 4°C with 5 μl of protein A:protein G Dynabeads (Thermo Fisher Scientific; 1:1) and 0.5 μl of the following antibody: H3K36me2 (C15310127, Diagenode; or 61019, ActiveMotif), H3K36me3 (ab9050, Abcam), or H3K27me3 (C15410069, Diagenode). Antibody-bond chromatin was washed twice in low-salt buffer [20 mM tris-HCl (pH 8.0), 2 mM EDTA, 150 mM NaCl, 1% Triton X-100, and 0.1% SDS] followed by two washes with high-salt buffer [20 mM tris-HCl (pH 8.0), 2 mM EDTA, 500 mM NaCl, 1% Triton X-100, and 0.1% SDS] and eluted in 0.1 nM NaCO_3_ and 1% SDS at 65°C for 1 hours. DNA was purified using SPRI beads (Omega) and used for paired-end library construction (NEBNext). Libraries were sequenced (150-bp paired-end) on the NovaSeq 6000 platform. Reads were clipped to 100 bp using Fastp (https://doi.org/10.1002/imt2.107) and then aligned using GenPipes’ chip.py pipeline (*57*). Visualization tracks were prepared using DeepTools bamCoverage (*58*).

### Quantitative reverse transcription PCR

Total RNA was isolated using RNA-Bee reagent (Tel-Test). Contaminating DNA was removed with the DNA-free DNA Removal Kit (Ambion). cDNA was prepared from 400 ng of total RNA using the SuperScript III Random Primer Synthesis kit (Invitrogen) for the quantitative PCR assays as we did earlier (*17*). The primer sequences are listed in table S1.

### Sequenom allelotyping

To measure the portion of each parental allele in the total transcript levels, multiplex Sequenom (now Agena Bioscience) allelotyping assays (*59*) were used. These assays are based on SNPs that distinguish between the inbred JF1/MsJ (JF1) and 129S1 (129) mouse strains. Each “unextended” primer (UEP) abuts a SNP in a target transcript, and the incorporating nucleotide differs in molecular mass between the parental alleles. The abundance of the extended UEP is quantified by mass spectrometry. Amplified cDNA samples were spotted onto a 384 SpectroCHIP Array (Agena Bioscience). Automated spectra acquisition was performed in a MassArrayCompact mass spectrometer (Sequenom) using the Spectroacquire program (Sequenom) and was analyzed by MassArray Typer v3.4. RNA-mixing standards were routinely run to verify linear response in measured versus input allele-specific transcription: For example, total RNA from JF1 and 129 embryos was mixed indifferent percent ratios (0:100, 10:90, 30:70, 50:50,70:30, 90:10, and 100:0) before cDNA preparation and Sequenom allelotyping. A 50:50 RNA mix was used for RNA skew correction. The percentage of transcription of each allele in the total expression was calculated at each given SNP. DNA samples were used from each embryo to verify the heterozygous genotype at each SNP. The UEP and PCR primer sequences are listed in table S2.

### Bisulfite sequencing of germ cells

A total of 2000 FACS-sorted male fetal germ cells were lysed with proteinase K solution and converted with EZ DNA methylation kit (D5001, ZYMO) according to the manufacturer’s instructions. PCR was performed using the genotype-specific forward primers and the shared reverse primer (table S3) and Zymo Taq hot start polymerase (catalog no. E2002, ZYMO) to amplify the converted DNA. The purified PCR fragment was ligated into pGEM-T vector (catalog no. A3600, Promega). Sanger sequencing of 15 independent colonies per region was performed by GENEWIZ. The QUMA online software was used for methylation analysis. Colonies that had identical pattern of the occasional incomplete conversion were excluded from the displays.

### Multiplex bisulfite sequencing

Fetal kidney DNA was purified by Proteinase K digestion, phenol-chloroform extraction, and ethanol precipitation. The EZ DNA methylation kit (D5001, ZYMO) was used for the bisulfite conversion according to the instructions of the kit. Multiplex PCR was performed with indexed primers (table S4) and Zymo Taq hot start polymerase (E2002, ZYMO) to amplify the converted DNA. The amplified DNA fragments were resolved using a 1% agarose gel, and the DNA was isolated from the gel slices using Monarch DNA Gel Extraction Kit (catalog no. T1020S). Fifty nanograms of each fragment was amplified with different index primers as listed below for Amplicon-EZ sequencing by GENEWIZ.

### Bioinformatics analysis of multiplex bisulfite amplicon sequencing

Reads were trimmed using TrimGalore v0.6.0 (https://github.com/FelixKrueger/TrimGalore) (*53*) with default settings to remove low-quality bases and Illumina adapters. Demultiplexing was done using CutAdapt v3.2 (*53*). The primer sequences, including the barcode sequences, were provided as anchored 5′ adapters using the “-g” and “-G” parameters, requiring no mismatches and no indels (“-e 0 --no-indels”). To account for read pairs where the barcode adapters were on the opposite read, the unmatched reads from the first CutAdapt run were processed with CutAdapt again using the same settings, except R1 and R2 were specified in reverse (R1 as R2 and vice versa). Last, the R1 and R2 reads from the two rounds of demultiplexing were concatenated together for each sample. Reads were aligned to the corresponding target sequence, padded with five N’s on each end to avoid issues during methylation context inference by the aligner, using Bismark (*60*) v0.23.0 with the parameters, “--non_directional” and “--local.” Per-CpG methylation percentages were obtained using the “bismark_methylation_extractor” script in Bismark with the parameters, “--bedGraph” and “--paired-end.”
