# Supplementary material for: Establishment of paternal methylation imprint at the H19/Igf2 imprinting control region
Source: Sci Adv. 2023 Sep 6;9(36):eadi2050. doi: 10.1126/sciadv.adi2050 (PMC10482337; doi:10.1126/sciadv.adi2050)
Supplement: Supplementary file 1 — Figs. S1 to S8 Tables S1 to S4 [file sciadv.adi2050_sm.pdf]

Science Advances
AAAS

Supplementary Materials for

Establishment of paternal methylation imprint at the *H19/Igf2* imprinting control region

Ji Liao *et al.*

Corresponding author: Piroska E. Szabó, piroska.szabo@vai.org

Sci. Adv. **9**, eadi2050 (2023)
DOI: 10.1126/sciadv.adi2050

This PDF file includes:

Figs. S1 to S8
Tables S1 to S4

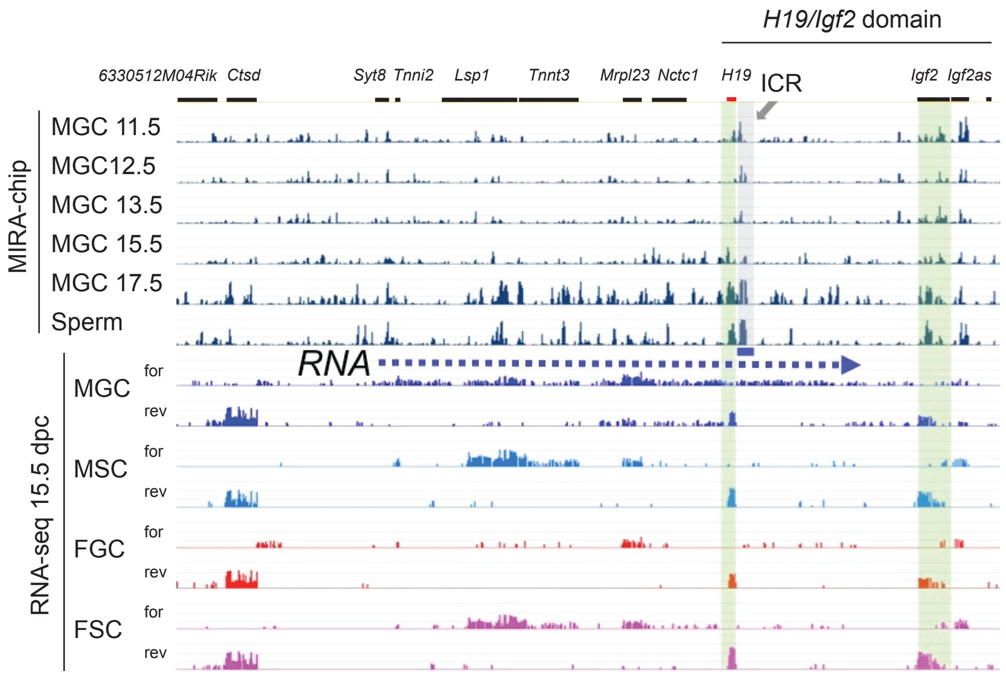

Figure S1. Broad low-level RNA transverses the *H19/Igf2* ICR in mouse prospermatogonia at the time of de novo DNA methylation.
MIRA-chip DNA methylation mapping results and RNA-seq results as done earlier (*4*) are displayed in the Signalmap browser (Nimblegen). The DNA methylation signals of MIRA versus input DNA were plotted as $-\log_{10}$ p value scores ranging from 0 to 8.4. The total RNA reads were evenly scaled to 50 M and are depicted in $\log_2$ scale ranging from 0 to 15. The transcripts are marked at the top, and the *H19/Igf2* domain is indicated with its imprinting control region (ICR). MIRA-chip was done on FACS-sorted fetal male germ cells (MGC), prospermatogonia, at the days of gestation as written in days post coitum (dpc), and in adult spermatozoa. Total stranded (forward and reverse, as marked) RNA-seq was done using FACS-sorted EGFP+ male germ cells (MGC), EGFP- male somatic cells (MSC), EGFP+ female germ cells (FGC), and EGFP-female somatic cells at 15.5 dpc. The broad low-level RNA that transverses the ICR at 15.5 dpc specifically in MGC is shown by a dotted blue line.

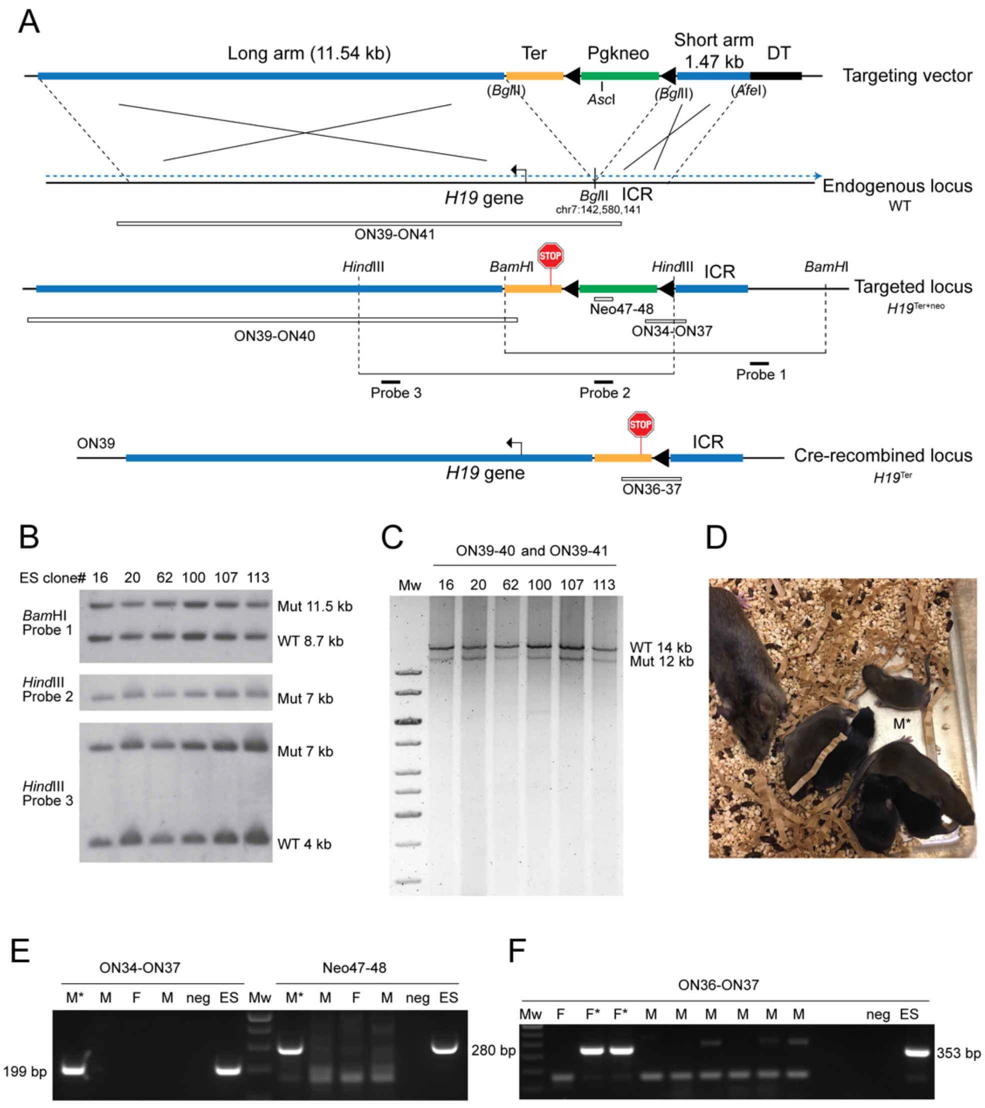

Figure S2. Generating terminator knock-in mouse models at the *H19/Igf2* ICR.
Gene targeting was done in ES cells to insert an RNA terminator cassette (Ter) next to the *H19/Igf2* ICR for truncating the fetal male germ cell specific broad low-level transcript (blue dashed arrow) before entering the ICR sequences. (**A**) Schematic maps depict the targeting vector, the endogenous locus, the targeted *H19*$^{\text{Ter+neo}}$ allele, and the Cre-recombined *H19*^{Ter} allele. The long and short arms of homology, Pgkneo positive selection cassette, diphteria toxin (DT) negative selection cassette, and two Lox*P* sites (black triangles), are marked. Southern probes and restriction fragments are indicated in the map of the targeted locus. Regions used for PCR genotyping are shown by open rectangles. Map is not to scale. (**B**) Southern blot hybridization results are shown of 6 correctly targeted ES cell clones with wild type (WT) and *H19*$^{\text{Ter+neo}}$ alleles (Mut). (**C**) PCR was used to confirm recombination along the long arm in the targeted ES cell clones. Clone 107 resulted in chimeras. (**D**) Its small size distinguishes a male agouti *H19*$^{\text{Ter+neo}}$ pup (M*) out of a male chimera, from its WT littermates. (**E**) Identification of a *H19*$^{\text{Ter+neo}}$ pup using PCR genotyping. The litter shown above was genotyped. Molecular weight marker (Mw), negative PCR control without DNA (neg), and positive PCR control (targeted ES cell DNA) are included. (**F**) Identification of *H19*^{Ter} pups using PCR genotyping.

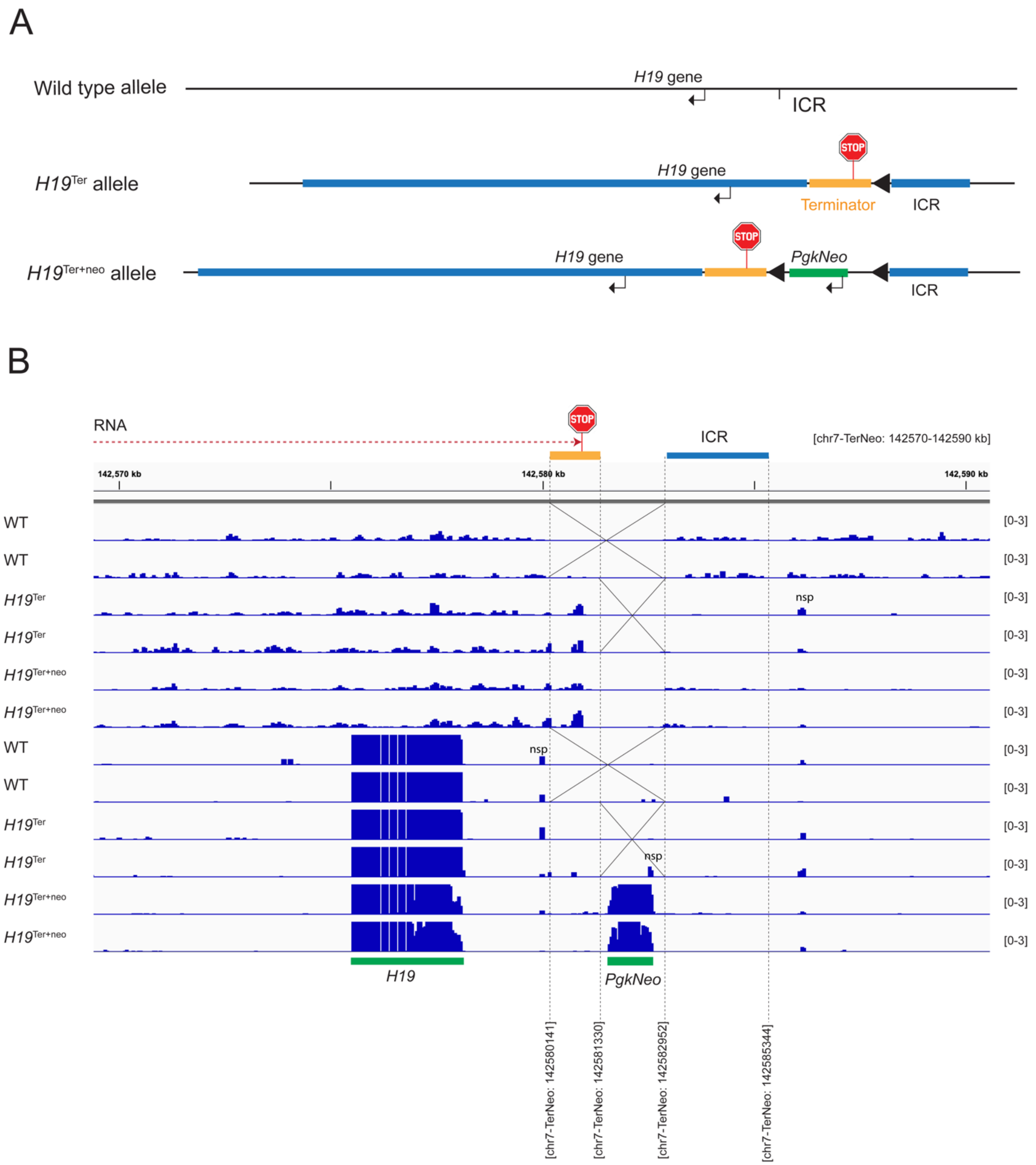

Fig. S3. The broad low-level RNA stops in the terminator cassette. (**A**) Schematic map of the wild type and mutant alleles. (**B**) IGV browser image of the deep total RNA-seq bigwig files in the forward (top six lanes) and reverse (bottom six lanes) direction after aligning to chromosome 7 including the Ter+neo insertion sequences (chr7-TerNeo). Coordinates of the Ter, and Pgkneo cassettes, and ICR are marked at the bottom. Regions that do not exist in the wild-type, or $H19^{Ter}/H19^{Ter}$ homozygous fetuses are crossed out in the respective chromosomes. Non-specifically (nsp) aligning peaks are marked. RNA-seq of purified prospermatogonia is shown in biological replicates from wild-type, $H19^{Ter}/H19^{Ter}$ and $H19^{Ter+neo}/H19^{Ter+neo}$ fetuses.

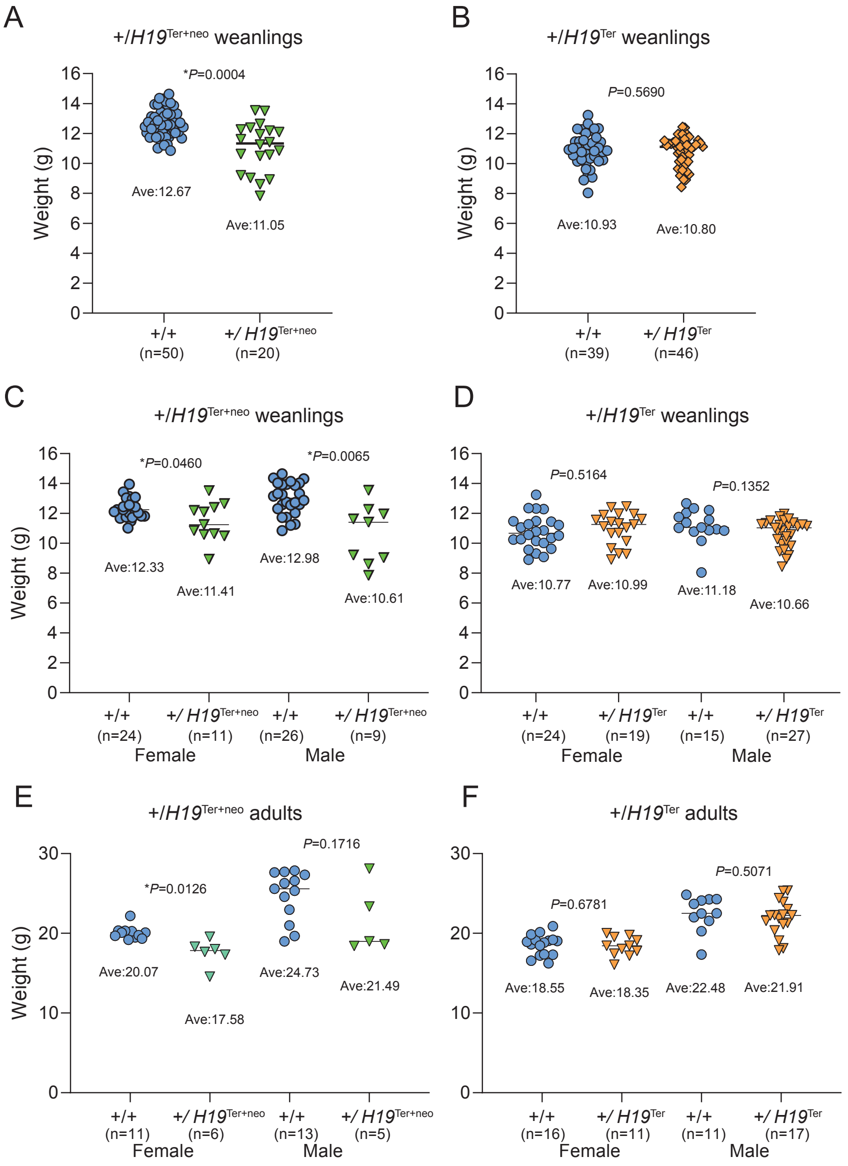

Figure S4. Offspring derived from epimutant prospermatogonia exhibit reduced growth after birth. (**A**) Weight distribution of the *H19*$^{+/}$ *H19*$^{Ter+neo}$ (n=20) and control *H19*$^{+/}$ *H19*$^{+}$ (n=50) pups obtained from total of 15 litters at the time of weaning at 21 days after birth. Mutant males were crossed with 129S1 wild type females to obtain these pups. The average values and the significance of difference between genotypes (*P*-value calculated by 2-tailed T-Tests) are marked. Note that the weight of the mutant pups is significantly reduced compared with WT siblings, and the number of mutant pups much is less than expected. (**B**) Weight distribution of the *H19*$^{+/}$ *H19*^{Ter} (n=46) and control sibling *H19*$^{+/}$ *H19*$^{+}$ (n=39) pups obtained from total of 12 litters at the time of weaning. (**C**) Weight distribution of the female and male *H19*$^{+/}$ *H19*$^{Ter+neo}$ and control *H19*$^{+/}$ *H19*$^{+}$ pups obtained from total of 15 litters at 21 days after birth. (**D**) Weight distribution of the female and male *H19*$^{+/}$ *H19*^{Ter} and control sibling *H19*$^{+/}$ *H19*$^{+}$ pups obtained from total of 12 litters at 21 days after birth. (**E**) Weight distribution of female and male *H19*$^{+/}$ *H19*$^{Ter+neo}$ and control *H19*$^{+/}$ *H19*$^{+}$ adults at 8 weeks after birth. (**F**) Weight distribution of female and male *H19*$^{+/}$ *H19*^{Ter} and control sibling *H19*$^{+/}$ *H19*$^{+}$ pups at 8 weeks after birth.

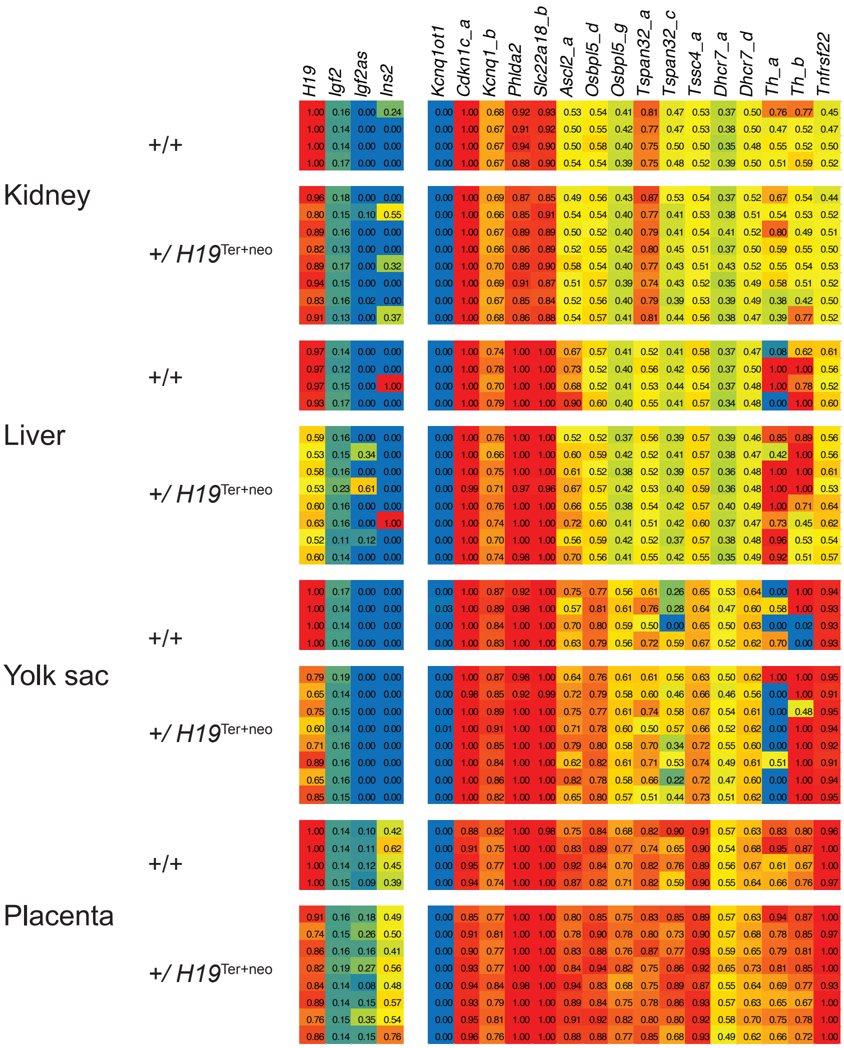

Fig. S5. Misexpression of imprinted genes is confined to the *H19/Igf2* imprinted domain along chromosome 7 in the offspring derived from $H19^{\mathrm{Ter+neo}}$ prospermatogonia. Allele-specific transcription of *H19*, *Igf2, Igf2as* and *Ins2* transcripts, together with seven transcripts in the neighboring *Kcnq1/Cdkn1c* imprinted domain in chr 7 is displayed in four fetal organs at 18.5 dpc. Sequenom allelotyping assays of uterus-mate biological replicate samples are shown. These were obtained by crossing a $H19^{+}/H19^{+}$ (JF1/MsJ) female with a $H19^{+}/H19^{\mathrm{Ter+neo}}$ (129S1) male. The numbers refer to the percent maternal allele expression in the total. The colors depict maternal allele-specific bias in red, paternal allele-specific bias in blue, and biallelic expression in yellow. Certain transcripts display parental-allele-specific transcription in specific organs. Note that the parental-allele-specific expression of only *H19* responded to the terminator mutation insertion in the kidney, liver, yolk sac and placenta organs.

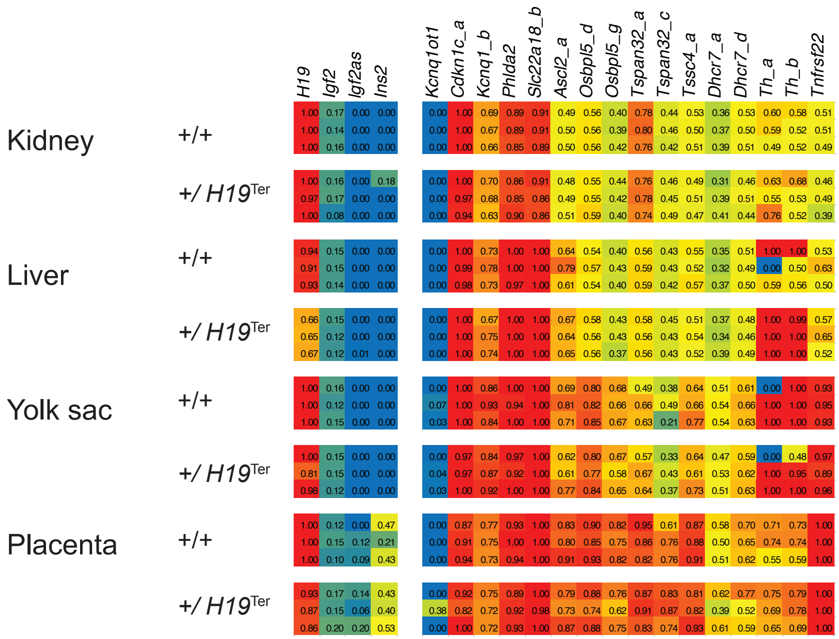

Fig. S6. Misexpression of imprinted genes is confined to the *H19/Igf2* imprinted domain along chromosome 7 in the offspring derived from *H19*^{Ter} prospermatogonia.
Allele-specific transcription of *H19*, *Igf2, Igf2as* and *Ins2* transcripts, together with seven transcripts in the neighboring *Kcnq1/Cdkn1c* imprinted domain in chr 7 is displayed in four fetal organs at 18.5 dpc. Sequenom allelotyping assays of uterus-mate biological replicate samples are shown. These were obtained by crossing a *H19*$^{+}$/*H19*$^{+}$ (JF1/MsJ) female with a *H19*$^{+}$/*H19*^{Ter} (129S1) male. The numbers refer to the percent maternal allele expression in the total. The colors depict maternal allele-specific bias in red, paternal allele-specific bias in blue, and biallelic expression in yellow. The parental-allele-specific expression of only *H19* responded to the terminator mutation insertion, most notably in the liver organ.

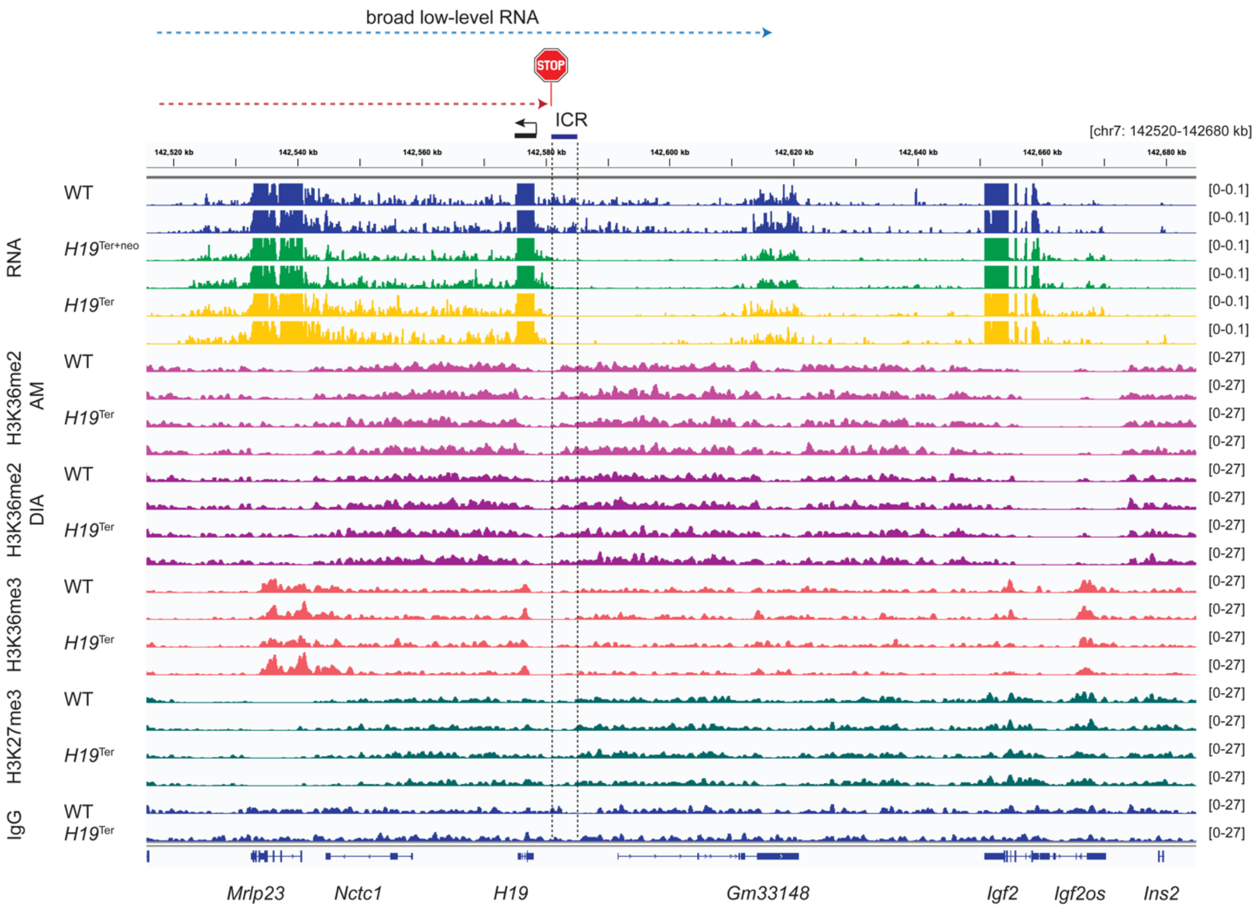

Fig. S7. The chromatin landscape of the *H19/Igf2* ICR and its surroundings is unchanged in 15.5 dpc prospermatogonia in response to the terminator cassette insertion. ChIP-seq results are shown in biological replicates of 15.5 dpc purified prospermatogonia from wild-type and *H19*^{Ter}/*H19*^{Ter} homozygous fetuses. Antibodies and samples are marked to the left. Other details are marked according to Figure 1.

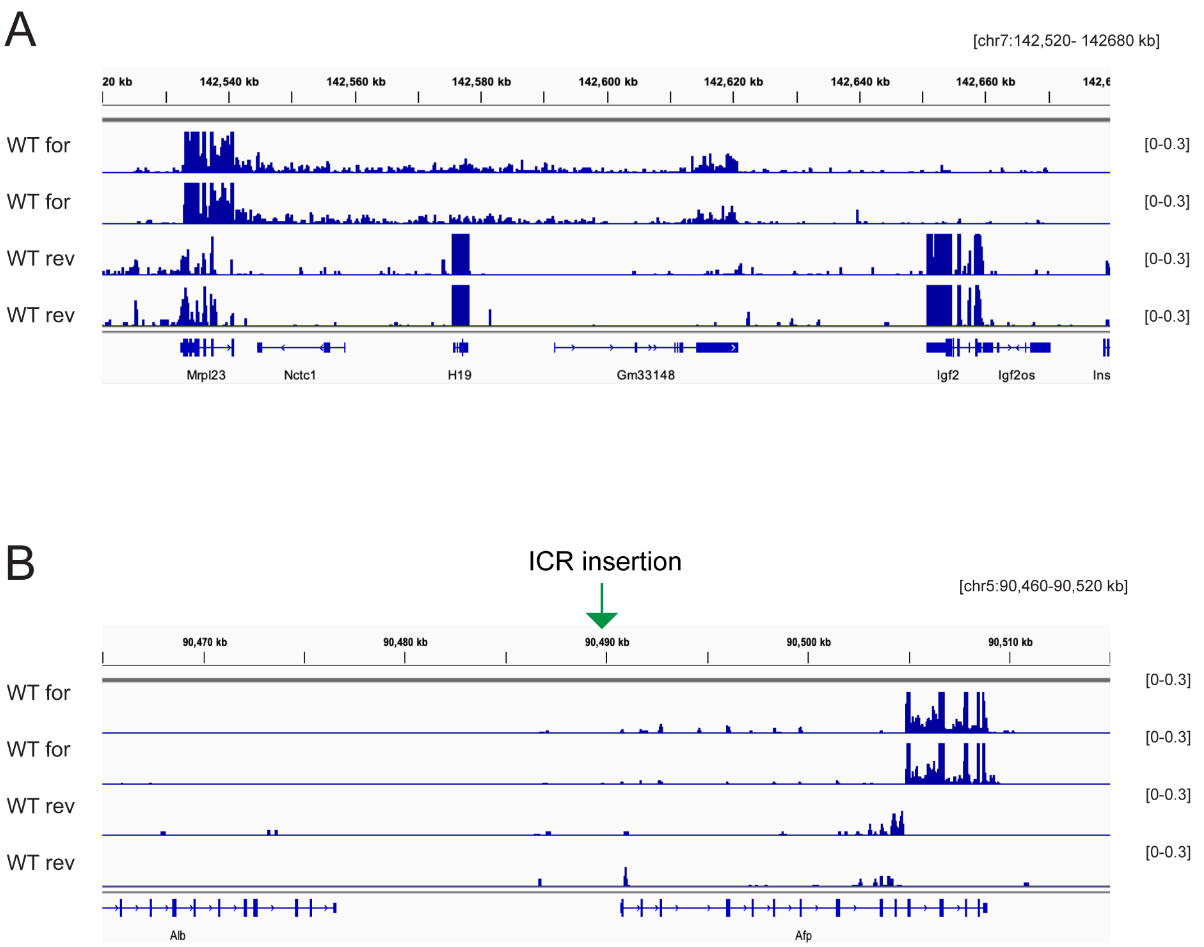

Fig. S8. Broad low-level transcription is not observed in prospermatogonia across the *Afp* promoter, where de novo methylation of an inserted ICR does not take place. Genome browser view of normalized total RNA-seq bigwig files in the forward (top two lanes) and reverse (bottom two lanes) directions. Biological replicates of 15.5 dpc purified prospermatogonia from wild-type (WT) fetuses are shown. Genes are marked. (**A**) *H19/Igf2* domain and surrounding regions is shown for reference. (**B**) *Afp* gene and its upstream region. The arrow shows the insertion site at -0.9 kb from the *Afp* TSS.

Table S1. Q-RTPCR Primers.

Igf2 exon 2-3 HEX	CCTTCAAGCCGTGCCAACCGTCGC
Igf2 exon 2-3 U	GGACCGCGGCTTCTACTTC
Igf2 exon 2-3 L	AGCAGCACTCTTCCACGATG
H19 exon 4-5 FAM	TGCCTCAGGAATCTGCTCCAAGGTG
H19 exon 4-5 U	CTGAATCAAGAAGATGCTGCAATC
H19 exon 4-5 L	GGTGCTATGAGTCTGCTCTTTC
Gapdh exon 5-6 Cy5	CGTGCCGCCTGGAGAAACCTGCC
Gapdh exon 5-6 U	AATGTGTCCGTCGTGGATCTG
Gapdh exon 5-6 L	CAACCTGGTCCTCAGTGTAGC

Table S2. Sequenom Primers.

Transcript/SNP_ID
2nd-PCRP
1st-PCRP
UEP_SEQ
Ascl2(a)_chr7:142967094
ACGTTGGATGCTTCATATTTTCAGTAGAG
ACGTTGGATGGGACTTGGAAATTTCTCAGG
ggcTATTTTCAGTAGAGTCCTACA
Cdkn1c(a)_chr7:143458462
ACGTTGGATGCCCCACACATTCATCTTCAG
ACGTTGGATGCATTTCGACTGTCTGGTCAC
tcgTCAGTTAGCTTACAGTGTCCCC
Dhcr7(a)_chr7:143837878
ACGTTGGATGATGGCATGTGACCAGTACAG
ACGTTGGATGTGTCTTGGCCCAAATGTCTG
tgcgCCCTGACTGCCCCCG
Dhcr7(d)_chr7:143847130
ACGTTGGATGGATGGTTGGTCATTCGGAAG
ACGTTGGATGGTACTTGGTGTACCACCCTG
GGTCATTCGGAAGATATAGTA
H19_chr7:149761651
ACGTTGGATGTTGCCCTCAGACGGAGATG
ACGTTGGATGGCTTTGAGTCTCTCCGTATG
AGCATTGCCAAAGAGG
Igf2_chr7:149836880
ACGTTGGATGACAGGTGACATATGGCGTTC
ACGTTGGATGGTCTCTTCCCTACTGTCTTC
ACAAAGGGAGGGACC
Ins2_chr7:142679601
ACGTTGGATGGCTAAGACCTCAGGGACTTG
ACGTTGGATGATAGGCTGGGTTGAGGATAG
TCAGGGACTTGAGGTAGGATAT
Kcnq1(b)_chr7:143426295
ACGTTGGATGTGATATGACAGCCTAGCTCC
ACGTTGGATGAAGTTCTAGTTCCCACTGGC
TGGCCTGGATCTGGATAT
Kcnq1ot1_chr7:150427528
ACGTTGGATGGGACACATGCATGCATCTGA
ACGTTGGATGTATGTTCCAGAGTTGTTGGG
CATGCATCTGATGATTTTTA
lgf2as ACGTTGGATGATCTCTAGCACAGGAGCATC
ACGTTGGATGGACTAGACTTCCTAGCCTTG
ggagCAGAGCTCCTGCAAAT
Osbpl5(d)_chr7:143689208

ACGTTGGATGGTTACTTCACCTACATCTGC
ACGTTGGATGTGGGCACTTCTCTTGCTTAC
ggccCCTACATCTGCCCTACA

Osbpl5(g)_chr7:143704576
ACGTTGGATGGACCTGTTCCACATAGGTAG
ACGTTGGATGAAGACCAACGAGAGTGGGAG
CCCACCAGGGCTGTC

Phlda2_chr7:143502389
ACGTTGGATGTGGTTTTCCCGGAGAAGAGG
ACGTTGGATGAAGCGAAGCGACAGCCTGTT
CAGCGCTTCTTCTTCCA

Slc22a18(b)_chr7:143476224
ACGTTGGATGTAGGTGAGTATGATGATCCC
ACGTTGGATGAGGACTCTGGATACTGTCTG
ATGATGATCCCCTGCCG

Th(a)_chr7:142896976
ACGTTGGATGTGGAGTACTTTGTGCGCTTC
ACGTTGGATGTGCGCACATCGTCAGACAC
gcgcCCTGGCTGCCCTCCTCAGT

Th(b)_chr7:142898143
ACGTTGGATGTCCCCAAGGTTCATTGGACG
ACGTTGGATGTCCAATGGGTTCCCAGGTTC
gtcgCATCGAGGATGCCCG

Tnfrsf22
ACGTTGGATGTTTCCTGATGTGCTGAGGAG
ACGTTGGATGCCAAACCTGGAATTCACCTC
gcttAGGAGAAGCAAATGGTTAG

Tspan32(a)_chr7:143005838
ACGTTGGATGGTTCCTGTGCACCAAGGAAG
ACGTTGGATGAGTTGCAGAGGCCCCTCAC
gggcaTAAGAAGCCCAGACCCTA

Tspan32(c)_chr7:143018812
ACGTTGGATGCTGCTAGACCCAATTGTCAG
ACGTTGGATGGTGCACATGTTTGCTAACTG
GACCCAATTGTCAGCAAACAC

Tssc4(a)_chr7:143070953
ACGTTGGATGACAATTCCCACAGTCTCTGC
ACGTTGGATGATCACTCTACCCCTCACCTG
acatACAGCCCACACCTTC

Table S3. Bisulfite Sequencing Primers.

H19TerNeo BS F1	GTTTTAGATTGTTTTGGGAAAAG
H19Ter BS F1	AAGATTTTATTAAAGTAGTTGGGATAG
H19 WT BS F1	TAGTTTTTGTTTTTATGGTTATGG
H19ICR BS R1	CATAACATTCAATAATTCATAAAAATCA

Table S4. Multiplex Bisulfite Sequencing Primers (in different combinations).

Indexed (index in bold) common reverse primers inside of the ICR:

503-H19ICR BS R1	AT**CCTATCCT**CATAACATTCAATAATTCATAAAAATCA
504-H19ICR BS R1	AT**GGCTCTGA**CATAACATTCAATAATTCATAAAAATCA
505-H19ICR BS R1	AT**AGGCGAAG**CATAACATTCAATAATTCATAAAAATCA
506-H19ICR BS R1	AT**TAATCTTA**CATAACATTCAATAATTCATAAAAATCA
507-H19ICR BS R1	AT**CAGGACGT**CATAACATTCAATAATTCATAAAAATCA
508-H19ICR BS R1	AT**GTACTGAC**CATAACATTCAATAATTCATAAAAATCA

Indexed forward primers:

701-H19Ter+Neo BS F1	AC**CGAGTAAT**GTTTTAGATTGTTTTGGGAAAAG
702-H19Ter+Neo BS F1	AC**TCTCCGGA**GTTTTAGATTGTTTTGGGAAAAG
701-H19Ter BS F1	AC**CGAGTAAT**AAGATTTTATTAAAGTAGTTGGGATAG
702-H19Ter BS F1	AC**TCTCCGGA**AAGATTTTATTAAAGTAGTTGGGATAG
701-H19WT BS F1	AC**CGAGTAAT**TAGTTTTTGTTTTTATGGTTATGG
702-H19WT BS F1	AC**TCTCCGGA**TAGTTTTTGTTTTTATGGTTATGG
